# Missed opportunities for screening congenital syphilis early during pregnancy: A case report and brief literature review

**DOI:** 10.3389/fpubh.2022.1073893

**Published:** 2023-01-06

**Authors:** Lei-Wen Peng, Yu-Jie Gao, Ya-li Cui, Huang Xu, Zheng-Xiang Gao

**Affiliations:** ^1^Department of Laboratory Medicine, West China Second University Hospital, Sichuan University, Chengdu, China; ^2^Key Laboratory of Birth Defects and Related Diseases of Women and Children, Sichuan University, Ministry of Education, Chengdu, Sichuan, China; ^3^Department of Laboratory Medicine, Meishan Women and Children's Hospital, Alliance Hospital of West China Second University Hospital, Sichuan University, Meishan, China

**Keywords:** awareness, congenital syphilis, pregnancy, prevention, *Treponema pallidum*, prenatal screening

## Abstract

Congenital syphilis is a significant public health problem. Pregnant women infected with Treponema pallidum present with various clinical manifestations, mainly including skin or visceral manifestations. The extensive clinical manifestations of *T. pallidum* infection mimic those of many other diseases during pregnancy, which may lead to delayed diagnosis and serious consequences. We report a case of fetal *T. pallidum* infection and premature delivery in a woman whose syphilis screening was negative at 16 weeks of gestation. Despite presenting to the dermatologist at 24 weeks of gestation with maculopapular rash which is usually associated with secondary syphilis, the diagnosis of syphilis was not considered. This case shows that even if early syphilis screening of pregnant women is negative, they may still get infected with *T. pallidum* later on in pregnancy. Therefore, in patients presenting with a rash without an obvious cause, *T. pallidum* infection should be excluded. The health status of patients' spouses should be assessed during pregnancy. Additionally, perinatal health education is necessary for women and their spouses during pregnancy. The abovementioned factors could reduce the probability of *T. pallidum* infection in pregnant women and their infants.

## Introduction

Syphilis is a chronic infectious disease caused by *Treponema pallidum*. In recent years, the incidence of syphilis has increased annually (1). Congenital syphilis is caused by *T. pallidum* infection and is generally transmitted vertically. If a pregnant woman with active syphilis is not effectively treated, *T. pallidum* can cross the placenta and infect the fetus *in utero*. Mother-to-child transmission of syphilis imposes heavy economic and medical burdens worldwide. The direct medical cost of mother-to-child transmission of syphilis is 309 million dollars per year (2). *T. pallidum* can infect women and be transmitted to the fetus at any stage of pregnancy, causing lesions in multiple organs and resulting in stillbirth, abortion, neonatal death, premature birth, low birth weight and other adverse pregnancy outcomes (3). Compared with uninfected pregnant women, pregnant women with syphilis are 12 times more likely to experience adverse pregnancy outcomes; those who receive treatment still have a 2.5 times higher risk than seronegative women (4). Among all global infectious diseases associated with stillbirth, fetal syphilis accounts for a large proportion of stillbirths (5). Therefore, early screening and treatment of pregnant women could significantly reduce the incidence of congenital syphilis. In this case study, we report a case of premature delivery and fetal congenital syphilis in a pregnant woman whose syphilis screening was negative in early pregnancy. It is important to recognize that congenital syphilis may still occur in countries with high prenatal screening rates, and women might be infected with *T. pallidum* between the time of testing during pregnancy and childbirth. Here, we review the literature on the epidemiology, transmission, clinical manifestations, diagnosis and prevention of *T. pallidum* infection and subsequently discuss the key information of this case.

## Case presentation

A premature baby boy was born at 34 weeks 1/7 days of pregnancy to a G2P2, 29-year-old mother. The infant had Apgar scale scores of 10, 10, and 10 at 1, 5, and 10 min, respectively. The birth weight was 2,200 g. The pregnant woman went to the hospital for prenatal care at 16 weeks 1/7 days of pregnancy, and serological tests for hepatitis B, hepatitis C, human immunodeficiency virus (HIV) and syphilis by chemiluminescent immunoassay (CLIA) were negative. The oral glucose tolerance testing (OGTT) results were as follows: fasting: 5.8 mmol/L, 1 h after meal: 11.7 mmol/L, and 2 h after meal: 11.8 mmol/L. A diagnosis of gestational diabetes was made, and diet control was recommended. At 24 weeks of gestation, the woman developed a generalized body maculopapular rash that was non-itchy. She went to the dermatology department of the local hospital, and the dermatologist confirmed that the HIV, hepatitis B and syphilis screening results were negative at 3 months of pregnancy. The maculopapular rash was considered to be related to pregnancy, and it subsided without treatment. The pregnant woman went to the hospital for prenatal care at 32 weeks 4/7 days of pregnancy, and the treponemal serological test (CLIA) was positive. The toluidine red unheated serum test (TRUST) titer was 1:16. Subsequently, her husband was screened for syphilis. The treponemal serological test (CLIA) was positive, and the TRUST titer was 1:32. The woman reported that she had unprotected sexual behaviors with her husband during pregnancy. Both individuals were treated with 2.4 million U of benzathine penicillin by intramuscular injection every 7 days for a total of three cycles.

**Figure 1:**
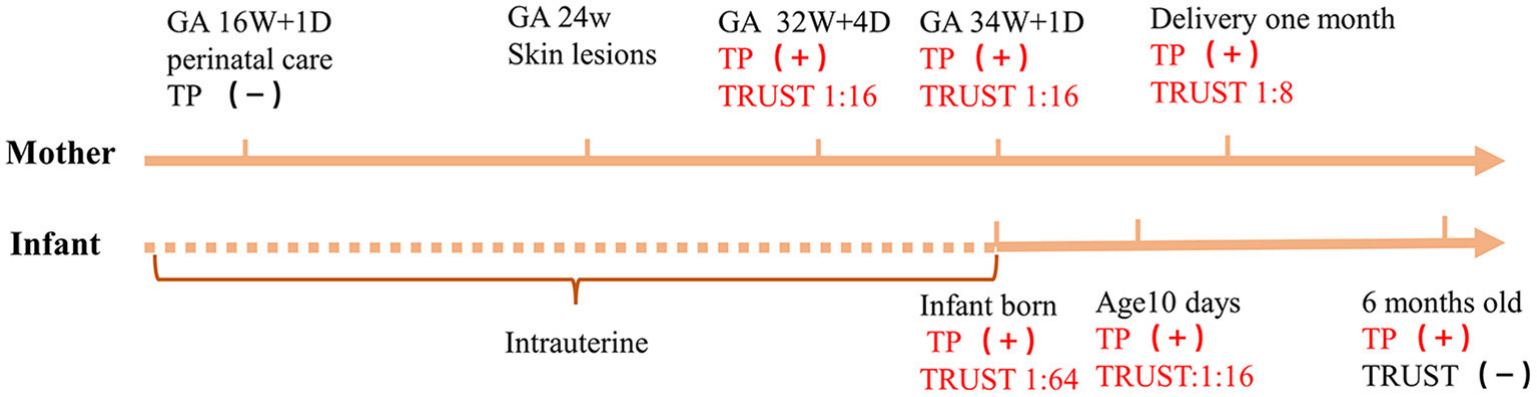
Timeline of the newborn and his mother. **+**, indicates a positive result; **−**, indicates a negative result; TP, treponemal serological test; GA, gestational age; 16W + 1D = 16 weeks 1/7 days, 32W + 4D = 32 weeks 4/7 days, 34W + 1D = 34 weeks 1/7 days.

At 34 weeks 1/7 days of pregnancy, the pregnant woman presented with abnormal vaginal bleeding. She was diagnosed with threatened premature labor and placental abruption was suspected; subsequently, the newborn was delivered by cesarean section. After birth, the newborn was transferred to the Neonatology Department for further care and treatment. Physical examination revealed the following: temperature of 36.3 C, pulse of 132 beats/min, respiration rate of 45 breaths/min, strong cry, and no petechiae or ecchymosis on his skin. Physical examination of the lungs, heart and abdomen was normal. The muscle tension of his extremities was normal. The four main primitive reflexes, namely, the sucking reflex, grasp reflex, rooting reflex and Moro reflex, could be drawn out completely. The laboratory test results are shown in Table 1. Skull ultrasound showed no abnormalities. The infant had no clinical manifestations of neurosyphilis, a nervous system physical examination showed no abnormalities. The results of CSF white blood cell and total protein tests were normal. Therefore, CSF testing for syphilis was not performed. After admission, the infant received an intravenous penicillin G solution (50,000 units/kg per 6 h) for 10 days and was then re-examined for syphilis. The treponemal serological test (CLIA) was positive, the TRUST titer was 1:16, and he was discharged home with his parents. When the infant was 6 months old, his parents brought him to the hospital for follow-up. His growth and development indicators were similar to those of normal children. The treponemal serological test was positive, and the TRUST was negative. The clinical manifestations and syphilis detection timeline of the mother and the patient are shown in Figure 1. We obtained informed consent from the infant's parents for the publication of this case report.

**Table 1:** Laboratory test results.

**Laboratory test**	**Value**	**Reference**
**Complete blood count**		
White blood cells (× 109/L)	12.36	5.30–12.00
Neutrophil percentage (%)	38.00	9.4–30.4
Lymphocyte percentage (%)	56.70	55.6–82.6
Red blood cells (× 1,012/L)	3.89	5.10–5.20
Hemoglobin (g/L)	155.00	129–151
Platelets (× 109/L)	136.00	100–300
**Blood biochemistry**		
Total bilirubin (μmol/L)	68.90	2.00–210.00
Direct bilirubin (μmol/L)	22.50	< 10.00
Indirect bilirubin (μmol/L)	46.40	< 193.00
Alanine aminotransferase (U/L)	9.00	< 49.00
Aspartate aminotransferase (U/L)	38.00	< 40.00
Total protein (g/L)	46.00	46.00–70.00
Albumin (g/L)	29.70	28.00–44.00
Globulin (g/L)	16.30	18.00–26.00
Alkaline phosphatase (U/L)	123.00	108.00–212.00
Cholinesterase (U/L)	5,991.00	4,000.00–12,600.00
C-reactive protein (mg/L)	< 5.00	0.00–10.00
Glucose (mmol/L)	6.24	3.90–11.10
K+ (mmol/L)	5.27	3.50–5.50
Na+ (mmol/L)	140.40	132.00–146.00
Cl- (mmol/L)	97.20	99.00–110.00
Ca2+ (mmol/L)	1.61	1.10–2.67
Urea nitrogen (mmol/L)	4.22	3.20–8.20
Creatinine (μmol/L)	56.00	17.30–57.60
Uric acid (μmol/L)	547.00	137.00–488.00
Cystatin C (mg/L)	1.2	< 1.02
**Cerebrospinal fluid test**		
Total cell count (× 106/L)	5.00	0.00–30.00
White blood cells (× 106/L)	5.00	0.00–30.00
Red blood cells (× 106/L)	0.00	0.00–0.00
Glucose (mmol/L)	1.97	2.50–4.40
Cl- (mmol/L)	114.10	120.00–130.00
Total protein (g/L)	0.42	0.15–0.45
Cerebrospinal fluid culture	Negative	Negative
**Blood culture**	Negative	Negative
**Treponemal serological test**	Positive	Negative
**Toluidine red unheated serum test (TRUST)**	1:64	Negative

## Review and discussion

### Epidemiology

The World Health Organization (WHO) estimates that ~2 million pregnant women are infected with syphilis each year and that the incidence of congenital syphilis is ~700,000–1.5 million. Syphilis in pregnancy kills an estimated 650,000 fetuses and newborns in developing countries annually (6). In 2016, the global prevalence of maternal syphilis was 0.7%, with a total of 661,000 congenital syphilis cases worldwide, resulting in 355,000 adverse pregnancy outcomes. Among these adverse pregnancy outcomes, 203,000 (57%) occurred in pregnant women who received antenatal care but did not undergo syphilis screening, 55,000 (16%) occurred in women who underwent syphilis screening but did not receive treatment, and 23,000 (6%) occurred in women who underwent screening and received treatment (7).

According to the latest statistics released by the Centers for Disease Control and Prevention (CDC) in 2021, the incidence of congenital syphilis in the United States has increased dramatically since 2012. A total of 1,870 cases of congenital syphilis were reported in 2019. The rate of congenital syphilis infection in 2019 was 41% higher than that in 2018 and 477% higher than that in 2012. From 2015 to 2019, the incidence of congenital syphilis increased by 291.1%. Most cases were due to delayed or a lack of screening in pregnant women and delayed or inadequate treatment (8). In most European Union (EU)/European Economic Area (EEA) countries, the incidence and number of congenital syphilis infections have remained low due to effective national antenatal screening programs and control of syphilis transmission in heterosexual populations. In 2019, only 13 EU countries reported 72 confirmed cases of congenital syphilis, which was an increase of 6 from 2018 (9). In China, the number of congenital syphilis cases ranged from 2.6 cases per 100,000 live births in 2000 to 69.9 in 2013 (10). The incidence of syphilis in China has increased annually, from 30.9 cases per 100,000 in 2014 to 38.4 in 2019, with an average annual growth of 4.4%. China's National Health Commission has strengthened and improved prevention strategies targeting mother-to-child transmission of syphilis, and the incidence of congenital syphilis decreased significantly from 54.1 cases per 100,000 live births in 2014 to 11.9 in 2019 (11).

Developed countries, such as EU/EEA countries, and developing countries, such as China, have reduced mother-to-child syphilis transmission, as evidenced by the low incidence of congenital syphilis, through high coverage of prenatal screening and treatment of infected pregnant women (12). This indicates that appropriate antenatal screening programs and congenital syphilis case reporting systems are very effective and necessary in eliminating mother-to-child syphilis transmission and reducing the rate of congenital syphilis.

### Mother-to-child transmission

*T. pallidum* can enter the fetal blood circulation through the placenta and cause congenital syphilis. The risk of syphilis transmission to the fetus through the placenta is 60–80%, and the risk increases in the second half of pregnancy. Neonatal exposure to secretions, blood or genital lesions of women with syphilis during delivery could also result in *T. pallidum* infection. Congenital syphilis most commonly occurs in infants of infected women who have not received adequate treatment or any treatment. Mother-to-child transmission can occur at any time during pregnancy, and the risk of transmission is related to the maternal stage of infection, which is relatively high at 60% to 90% in untreated women with primary or secondary syphilis, 40% in women with early latent syphilis, and < 10% in women with late latent syphilis (13). Pregnancy syphilis can be divided into primary syphilis, secondary syphilis, tertiary syphilis and latent syphilis. A chancre often develops in primary syphilis. The most common symptom of secondary syphilis is a rash that is widely distributed on the skin and mucosa. The most common rash types are maculopapular (50–70%), papular (12%), macular (10%) and annular papular (6–14%). The rash typically appears on the palms and soles of the feet (14, 15). Tertiary syphilis mainly manifests as severe damage to the bone, skin, mucous membranes or other organs.

In this case, the mother had no syphilis-related clinical features before pregnancy, she denied a history of *T. pallidum* infection or other sexually transmitted diseases, and she had unprotected sexual behaviors with her husband during pregnancy. The pregnant woman went to the medical institution for prenatal care in early pregnancy and was screened for sexually transmitted diseases, including syphilis, and no abnormalities were found. At ~24 weeks of gestation, the pregnant woman presented with a non-itchy maculopapule with no obvious cause. She went to the dermatology department of the local hospital, and syphilis infection was not considered in the diagnosis. Although skin abnormalities may be one of the clinical manifestations of syphilis infection, the lesions of syphilis (which are known to be great imitators) are often mistaken for those of other infections, and early diagnosis may be hampered.

The clinical features of syphilis in pregnant women are the same as those in non-pregnant people. However, latent syphilis is most frequently reported in pregnancy, and ~70% of *T. pallidum*-infected pregnant women have latent syphilis (16). Therefore, it is difficult to identify and diagnose the disease depending only on the pregnant woman's medical history and clinical symptoms. In some countries, syphilis in pregnancy can be diagnosed and effectively treated in a timely manner through syphilis screening, which can prevent congenital syphilis. In this case, the pregnant woman did not visit a medical institution for antenatal care and syphilis screening at 28 weeks of pregnancy; thus, the opportunity to diagnose and treat the infection earlier was likely missed and increased the incidence of *T. pallidum* infection of the fetus and premature delivery. *T. pallidum* is easily transmitted to the fetus, leading to adverse pregnancy outcomes. This case illustrates that owing to the low rate of *T. pallidum* infection, syphilis may not be considered in the differential diagnosis when the patient has non-specific symptoms, such as rash. More importantly, for pregnant women with negative syphilis screening results in early pregnancy, it is less likely that *T. pallidum* infection is considered when they have non-specific symptoms (such as maculopapular rash) in the second and third trimesters. Therefore, clinicians should be aware that pregnant women may still be infected with *T. pallidum* after prenatal screening.

### Clinical manifestations of and diagnostic tests for congenital syphilis

Congenital syphilis is generally divided into early congenital syphilis, late congenital syphilis and latent congenital syphilis (17). Early congenital syphilis can present at any time within 2 years after birth; it usually presents in the neonatal period and rarely later than 3–4 months after birth. Most affected infants are asymptomatic at birth, and two-thirds of infants develop clinical symptoms at 3–8 weeks after birth. Neonates are often born prematurely, and infant symptoms include stunting, underweight, low-grade fever, anemia, thrombocytopenia, osteochondritis, nasal congestion, maculopapular rash, and iritis. Late congenital syphilis results in a variety of skeletal and dental defects as well as neural deafness and interstitial keratitis, which usually develops at 2 years of age (18). Latent congenital syphilis generally causes no symptoms and is diagnosed by only a positive serological test; moreover, the examination of cerebrospinal fluid is often normal. Because the clinical manifestations of congenital syphilis are diverse and often non-specific, even absent, the diagnosis is often missed.

The detection methods for congenital syphilis mainly include direct detection and serological detection. Direct detection includes dark field microscopy and *T. pallidum* fluorescence immunostaining methods. The advantages of these two methods are that they are simple and rapid, but they require high-quality clinical specimens, and a negative test cannot rule out *T. pallidum* infection. Currently, the abovementioned methods have been largely replaced by more sensitive nucleic acid amplification tests (NAATs). Placental tissue, amniotic fluid, neonatal mucus, cerebrospinal fluid and blood may be suitable sample types for NAATs (19). Compared to that of culture, sensitivity using Tpp47 targets was 75–100% in amniotic fluid, 60–75% in neonatal cerebrospinal fluid, and 67–94% in neonatal whole blood or serum (20). Although the detection of *T. pallidum* by NAATs is feasible, this method is expensive, and its sensitivity and specificity vary depending on the tested tissue and target gene; therefore, it is not widely performed in hospitals.

Serological testing is still the main laboratory method for the diagnosis of congenital syphilis. There are two types of tests: treponemal serological tests and non-treponemal serological tests. The specificity for treponemal serological tests is higher than that of non-treponemal serological tests. However, a positive test cannot be used to diagnose congenital syphilis because it cannot be determined whether IgG antibodies are of maternal origin or from neonates (21). When the fetus is infected by *T. pallidum*, B lymphocytes will generally produce anti-*T. pallidum* IgM antibodies after ~2 weeks, which are not able to cross the placental barrier due to their high molecular weight. With the development of antibody preparation technology, anti-*T. pallidum* IgM antibody tests have been gradually applied for laboratory diagnosis. The current detection methods include the 19S-IgM-fluorescent treponemal antibody absorption test, *T. pallidum* immunity blot assay and *T. pallidum*-IgM-enzyme-linked immunosorbent assay (22). If detected in neonatal blood or cerebrospinal fluid, congenital infection is considered, but a negative test does not exclude the diagnosis. Moreover, due to limitations of reagents, instruments and operating procedures, the above three tests have not been widely applied in hospitals. Non-treponemal serological tests mainly include the rapid plasma response (RPR) test, TRUST, and the venereal disease research laboratory (VDRL) test. To establish a diagnosis of congenital syphilis, the neonatal titer should be at least 4 times higher than the maternal titer on the non-treponemal serological test (21).

Congenital syphilis diagnosis requires a combination of maternal serological findings and treatment, comparison of maternal and neonatal non-treponemal serological titres, and clinical evaluation of the neonate. Different countries have different definitions and diagnostic criteria for congenital syphilis. In China, the diagnostic criteria for congenital syphilis are any of the following in children born to syphilis-infected mothers: 1. *T. pallidum* detection in skin/mucosal lesions and tissues in infants with early congenital syphilis by dark-field microscopy or silver staining or a positive *T. pallidum* nucleic acid test; 2. *T. pallidum* IgM antibody serological test positivity; 3. a positive non-treponemal serological test at birth, a ≥ 4-fold higher titer than the maternal titer, and treponemal serological test positivity; 4. non-treponemal serological test negativity at birth or a titer < 4-fold higher than the maternal titer, and a change in the test from negative to positive at the subsequent follow-up; alternatively, the titer increased with clinical symptoms, and the treponemal serological test was positive; and 5. treponemal serological tests remained positive throughout the first 18 months after birth (23). According to the 2021 sexually transmitted disease (STD) Prevention and Control Guidelines in the United States, congenital syphilis can be diagnosed by meeting criterion 1 or 3. A treponemal serological test is not recommended because passively transferred maternal antibodies can persist for as long as 18 months (8).

A commonality between the above two diagnostic criteria is a neonatal titer for the non-treponemal serological test that is 4-fold higher than the maternal titer. In addition to pathogen detection, this is a key indicator of congenital syphilis diagnosis. The newborn with early congenital syphilis had no typical symptoms after birth in this case. The diagnosis was mainly based on the mother's history of syphilis infection and serological testing. The treponemal serological test(CLIA) was positive, and the TRUST titer was 1:64, i.e., 4-fold higher than the mother's titer (1:16). Related studies have shown that neonatal non-treponemal serological test titres 4-fold higher than those of the mother are rare, with sensitivities ranging from 4 to 13% (21). Considering the abovementioned results, this newborn was diagnosed with early congenital syphilis.

### Prevention

No effective vaccine has been developed to prevent syphilis infection (24), but congenital syphilis is preventable through syphilis screening and treatment in early pregnancy. The WHO recommends that all women be tested at their first antenatal visit and again in the third trimester. If the result is positive, it is recommended that their partner also undergo syphilis screening and treatment. The United States CDC recommends that all pregnant women should be screened for syphilis at the first prenatal visit. If there are risk factors for syphilis infection (living in a community with high syphilis morbidity or behaviors conducive to syphilis acquisition during pregnancy) during pregnancy, syphilis screening should be performed again at 28 weeks of pregnancy and at delivery (25). The European CDC recommends that pregnant women should be screened for syphilis within the first 3 months of pregnancy. Retesting is recommended in late pregnancy (before 32 weeks of gestation) to allow sufficient time for effective treatment. If syphilis screening is not performed during pregnancy, it should be done at the time of delivery. An analysis of 25 published studies evaluating the effectiveness of infection screening and infection management interventions during pregnancy found that syphilis-focused interventions resulted in a significant 80% reduction in stillbirth rates compared to strategies to treat, detect and/or prevent other infections in pregnancy (26).

In China, a management system has been established to standardize maternal health care, which provides strong support for the prevention of syphilis in pregnancy and the control of congenital syphilis. Since 2015, China's National Health Commission has comprehensively carried out prevention strategies targeting mother-to-child transmission of HIV, syphilis and hepatitis B and provided free screening for HIV, syphilis and hepatitis B for pregnant women and intervention services for infected pregnant women and their children. It is recommended that the first prenatal care visit and syphilis screening be completed within the first 3 months of pregnancy. Pregnant women with high-risk factors for *T. pallidum* infection in late pregnancy (28 weeks) and at the time of delivery should undergo syphilis screening again; this process could prevent *T. pallidum* infection in the fetus in the second and third trimesters of pregnancy. A study showed that 500,000 pregnant women from Shenzhen (China) underwent syphilis screening and that the mother-to-child transmission rate of syphilis decreased significantly from 54/100,000 to 22/100,000 (27). Another study in China analyzed 23,879 pregnant women from 2009 to 2018 and found that the prevalence of syphilis among pregnant women decreased from 1.5 to 0.3% after the implementation of prenatal screening (28). The above two studies indicate that regular prenatal syphilis screening can greatly reduce the incidence of congenital syphilis.

In this case, the pregnant woman did not seek timely prenatal care and syphilis screening at 28 weeks of gestation after the first negative prenatal test. Syphilis screening was performed at 32 weeks 4/7 days of gestation, and the treponemal serological test (CLIA) was positive, with a TRUST titer of 1:16. Before the end of treatment, the fetus was delivered because of threatened premature delivery at 34 weeks 1/7 days of gestation, and congenital syphilis was found by laboratory testing. Studies have shown that missing the optimal window for diagnosing syphilis is mainly due to pregnant women having negative screening results at their first prenatal visit and not being rescreened at delivery (29) or because routine syphilis screening is not offered to pregnant women in some countries (30). If a pregnant woman lacks prenatal care awareness, even though the initial syphilis screening is negative, she may be infected with *T. pallidum* in the second half of pregnancy and may transmit it to the fetus. Considering that most pregnant women infected with syphilis have non-specific symptoms, it is necessary to strengthen prenatal health education, especially concerning sexually transmitted diseases. This will promote regular prenatal care visits and the detection and effective treatment of syphilis in a timely manner.

## Conclusion

The case presented in this article shows that mother-to-child transmission of syphilis still occurs in countries with high coverage rates of prenatal syphilis screening. The incidence of congenital syphilis is related to not only the syphilis infection rate but also the antenatal care coverage rate. The extensive clinical manifestations of syphilis infection mimic those of many other diseases during pregnancy, which may lead to delayed diagnosis and serious consequences. Therefore, the doctor should exclude the possibility of *T. pallidum* infection in pregnant women with a rash with no obvious cause, even if the early syphilis screening result was negative. Otherwise, this preventable infectious disease is easily overlooked. Additionally, it is very important to promote prenatal care, increase prenatal health awareness for pregnant women and their spouses, and encourage regular syphilis screening in all women during pregnancy. This could minimize the adverse influence of syphilis on pregnant women and their infants.

## Data availability statement

The original contributions presented in the study are included in the article/supplementary material, further inquiries can be directed to the corresponding author.

## Ethics statement

Written informed consent was obtained from the individual(s) legal guardian for the publication of any potentially identifiable images or data included in this article.

## Author contributions

Y-JG analyzed the data. L-WP wrote the manuscript. Z-XG revised the manuscript. Y-lC and HX collected the data. All authors contributed to the discussion of the article and agreed to be accountable for the content of the work.
